# Towards point-of-care diagnostics of neglected tropical diseases using nucleic acid amplification tests

**DOI:** 10.1007/s00216-026-06761-2

**Published:** 2026-08-31

**Authors:** Nicolina Priem, Pablo Rodriguez-Mateos, Harrison Waweru, Jesse Gitaka, Nicole Pamme

**Affiliations:** 1 Department of Chemistry, Stockholm University, Stockholm, Sweden; 2 Severo Ochoa Molecular Biology Centre (CSIC-UAM), Madrid, Spain; 3 Mount Kenya University, Thika, Kenya

**Keywords:** Neglected tropical diseases, Point-of-care, Diagnostics, Nucleic acid amplification

## Abstract

Neglected tropical diseases (NTDs) impose a substantial global burden, yet diagnostic innovation for these conditions has historically been lower than for other major infectious diseases. Addressing this diagnostic gap is a prerequisite for their elimination, as recognised by the World Health Organization (WHO). This critical review evaluates the current scope of point-of-care nucleic acid amplification tests (POC NAATs) through a systematic analysis of 39 studies. This is achieved via a breakdown of the nucleic acid amplification workflow and associated implementation barriers with a focus on operational feasibility rather than analytical metrics such as sensitivity and specificity. The reviewed publications showed several reoccurring themes such as simplified extraction methods, integrated workflows on platforms or devices, smartphone apps for interpretation, reducing cold-chain dependence, and electricity-independent workflows. However, several technical, analytical, and implementation barriers were also identified which hinder the transition from proof-of-concept prototypes to field-deployable tools that meet the criteria set up by the WHO for POC tests. The review showcases that a majority of current NAAT developments for NTDs do not yet fulfil the WHO criteria for POC application in resource-limited settings. Whilst there are opportunities for improvement as well as for repurposing NAAT platforms for other diseases, careful examination of all steps from sample collection to mature implementation in the field is required to achieve the aim set up by the WHO.

**Graphical Abstract:**

**Figure:**
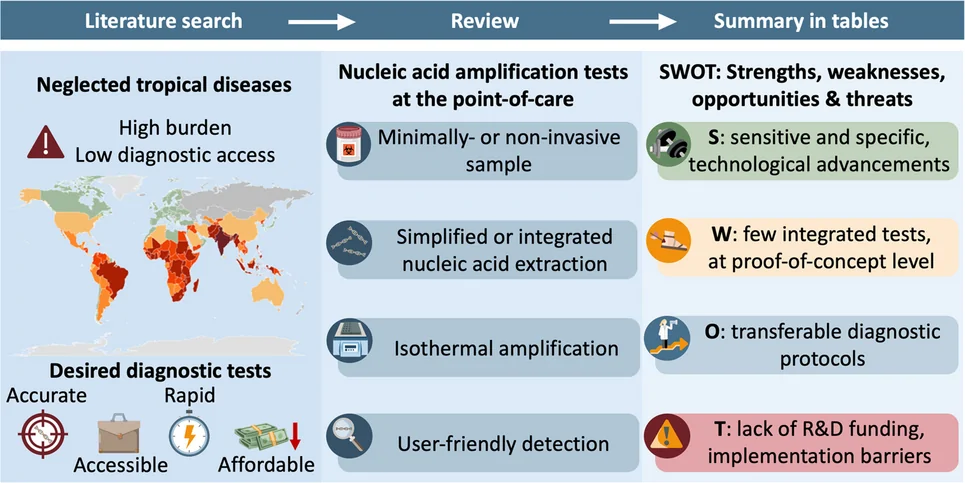

## Introduction

Neglected tropical diseases (NTDs) are a diverse group of parasitic, bacterial, and viral conditions with severe health and economic impact that predominantly affect vulnerable, marginalised populations. Public health control is inherently complex since many of these diseases are vector-borne or have animal reservoirs as well as complex life cycles which complicates intervention [1]. Consequently, in regions with limited access to clean water, sanitation, and healthcare, these diseases thrive [2]. This can in turn lead to a perpetuated cycle of poverty characterised by poor educational and professional outcomes as well as stigma and social exclusion. Historically, these conditions have been recognised to affect the quality of life in 149 countries but have despite this received disproportionately low attention on the global health agenda and funding compared to diseases such as malaria, even though NTDs represent a substantial burden [3–5]. Currently, these diseases affect over 1 billion people and are related to over 100,000 deaths per year, and a lack of access to timely treatment has left hundreds of millions severely disabled or debilitated despite many NTDs being treatable [1, 4, 6].

Over the past decade, progress has been made to control and eliminate these diseases with a reduction in disability-adjusted life years from 17 million in 2015 to 14 million in 2021 and people requiring intervention from 1.8 billion in 2015 down to 1.5 billion in 2023 [6]. However, this number of afflicted individuals remains high and declining prevalence in some regions has rendered previous solutions increasingly inadequate. Moreover, challenges such as lack of disease awareness, delayed healthcare-seeking behaviour, migration, climate change, and antimicrobial resistance remain and exacerbate the problem [1, 3, 7].

To address this, the World Health Organisation (WHO) has identified diagnostic innovation as a priority area. As a foundational step, diagnostic tests have a major influence on healthcare decisions, yet their development has not received adequate funding. Consequently, control of NTDs has been attempted using other strategies, such as mass drug administration (MDA) [1, 8]. These programmes target NTD-endemic populations which makes them time-efficient, but it is a presumptive approach that provides limited information in comparison to accessible diagnostics. With such limited data, prolonged reliance on MDA creates surveillance blind spots that leave millions of people as missed cases without treatment, whilst simultaneously forcing programmes to be extended and driving, for example, up to 50% excessive use of medicines for lymphatic filariasis. Despite the cost of these prolonged programmes, only about 5% of the funding for research and development investments for NTDs goes to new diagnostic tests. With this low funding, benefits such as accelerated elimination, reduced morbidity, lower programme costs, disease mapping, screening, surveillance, and monitoring are not achieved, which complicates decision-making.

### Conventional diagnostics of neglected tropical diseases

The conventional diagnostic methods for neglected tropical diseases show a trade-off between affordability and the accuracy, sensitivity, and specificity required for reliable diagnostic tests [1]. These methods include microscopy and serological, molecular, and culture-based assays as well as clinical presentation [9]. Whilst each method shows advantages, their inherent disadvantages make them insufficient for NTD diagnostics in low- and middle-income countries (see Table 1).

**Table 1:** Conventional methods used for diagnostics of NTDs with their respective advantages and disadvantages

Standard method	Advantages	Disadvantages	Ref
Microscopy	+ Simple or no sample preparation + Few consumables needed	– Requires trained personnel – Low sensitivity	[1, 10, 11]
Serological (e.g. ELISA)	+ Rapid + High throughput	– Limited use for early infections – Low specificity and sensitivity	[1, 9]
Molecular (e.g. PCR)	+ High specificity and sensitivity + Useful for early detection	– Requires sample preparation – Cost/test usually high	[1, 9, 11]
Culture	+ Conclusive identification + Essential test according to WHO	– Long time to result – Requires biosafety measures	[1, 12]
Clinical presentation	+ Immediate + Equipment-free	– Low specificity and sensitivity – Prone to bias and overdiagnosis	[1, 10]

This has become a particular issue since the progress that has been made to control these diseases has resulted in lower infection loads, which has rendered the low-sensitivity tests inadequate [10]. Moreover, these tests require specialised personnel and equipment to varying extents, which is challenging to implement at decentralised health clinics in resource-limited settings where the need for diagnostic tests is high [11, 12]. Since the priority of the WHO is for new diagnostic tests to be accessible, applicability at the Point-of-Care (POC) becomes a key criterion. This, however, comes with specific challenges that are, as of yet, not met for most of the NTDs [4].

### Identified diagnostic needs

Each NTD has disease-specific priorities defined by the WHO, which include the availability of effective and affordable diagnostics for eradication, interruption of transmission, elimination as a public health problem, or disease control [1, 4]. According to the 2025 WHO global report, none of the 26 recognised NTDs has existing diagnostic tests that facilitate reaching the established target [6]. Furthermore, approximately 31% of these conditions are flagged as requiring critical action to reach the 2030 objectives. To aid in the development of diagnostic tests with desired characteristics, the WHO has established target product profiles (TPPs) for conditions including Buruli ulcer [12, 13], human African trypanosomiasis [14, 15], leprosy [16], leishmaniasis [17, 18], lymphatic filariasis [19], mycetoma [20], onchocerciasis [21], scabies [22], schistosomiasis [23], soil-transmitted helminths [24], trachoma [25], and yaws [26]. Whilst these TPPs vary in parameters such as sample type, species differentiation, and targeted population, several common technical requirements are shown across the group (see Table 2). The challenge for these tests lies in achieving high sensitivity and specificity in decentralised settings whilst also mitigating operational constraints such as restricted power and water access through innovative approaches.

**Table 2:** Summary of desired characteristics of diagnostic tests based on 14 TPPs. Ranges are presented for factors that have varying required stringency [13–26]

Factor	Field-based	Laboratory-based
Lowest infrastructure level	Ideal: “Zero-infrastructure” Minimum: “Minimum infrastructure”	Diagnostic testing laboratory
Lowest level user	Community health personnel or health workers	Laboratory technicians
Training requirements	< 0.5–2 days	< 5–14 days
Clinical sensitivity	> 60–99%	> 60–99%
Clinical specificity	> 90–99.9%	> 90–99.9%
Portability	High without specialised transport	No requirement
Instrument and power requirement	Self-contained kit, independent of mains power source	Plug-in power
Water requirement	Independent of water supply	Access acceptable
Operating temperature and humidity	15–40 °C 10–88% relative humidity	Controlled temperature and humidity acceptable
Maintenance and calibration	No maintenance or calibration	Once a year
Sample preparation	Transfer of sample to testing device	Transfer of sample to testing device
Ease of use	One timed step ≤ 3–10 user steps Unprotected external environment	≤ 5 timed steps ≤ 15 user steps
Detection and interpretation	Naked eye	Can be instrument-based
Time to result	< 0.5–1 h	< 48 h
Supplies needed	Included in kit	Included in kit
Storage conditions	No cold storage	Cold storage acceptable

### Point-of-care diagnostic tests

To achieve an accessible diagnostic test in resource-limited settings, where the main burden of NTDs occurs, the WHO describes the ideal characteristics of POC diagnostic tests with the acronym ASSURED (Affordable, Sensitive, Specific, User-friendly, Rapid and robust, Equipment-free, Deliverable to end-user) [4, 27, 28]. This was originally coined by the WHO Sexually Transmitted Diseases Diagnostics Initiative in 2003 but has since been applied to other diseases. To account for lessons learned and advances in technology over the years since the implementation of the criteria, additional factors of real-time connectivity, ease of specimen collection, and environmental friendliness have been suggested to an updated REASSURED criteria [28]. Recently, research dedicated to developing such tests has increased with growing attention to NTDs as well as multiple disease outbreaks during the twenty-first century, e.g. Ebola and COVID-19 [28, 29].

POC diagnostics that follow the REASSURED criteria show advantages and benefits not only for patients, but also for operators, medical practitioners, health services, and communities as a whole [28, 30]. These benefits include improved patient outcomes, reduced patient loss to follow-up, opportunities for patient education, timely clinical decisions, and reduction of unnecessary medicine consumption [30]. Some of these advantages stem from eliminating the need to transport the sample to a laboratory for a centralised test and waiting for the result to come back since the diagnosis through a POC test can be performed in a single clinical encounter which increases the chance of timely interventions [30, 31].

### Nucleic acid amplification tests

**Fig. 1:**
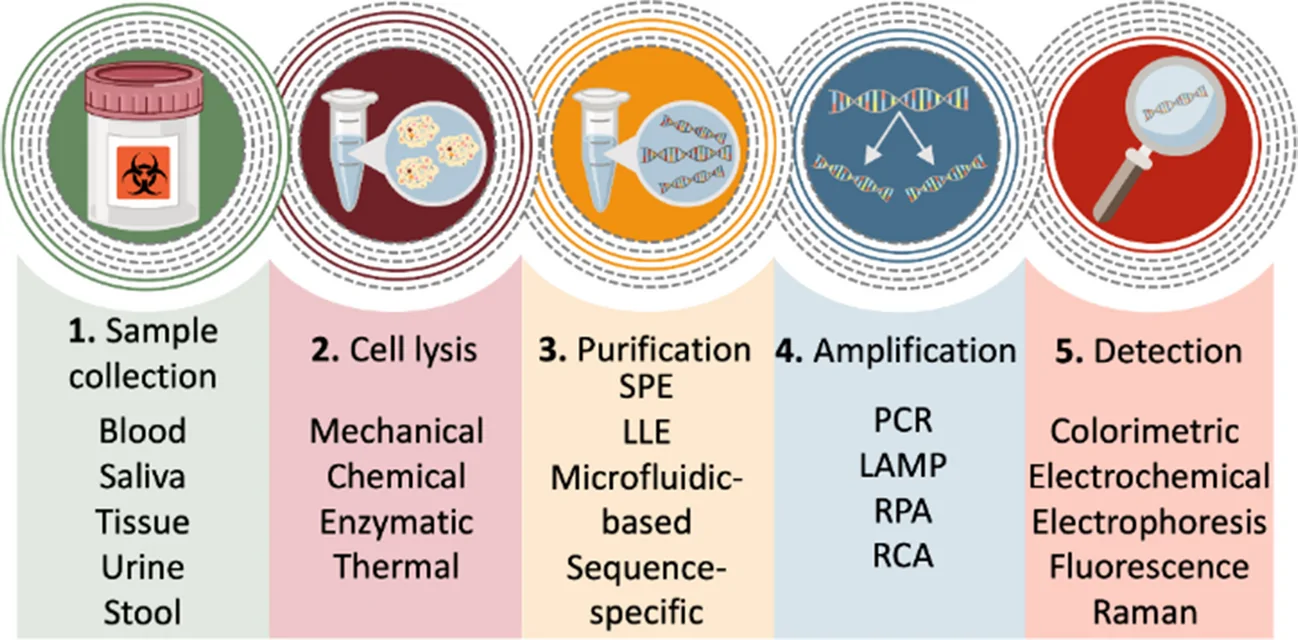
Stepwise procedure of a nucleic acid amplification test. The tests start with sample collection which should be minimally or non-invasive for POC tests. The sample is then usually subjected to several sample preparation steps that often start with cell lysis. The lysis step can be done in a number of different ways and should result in breaking the cell membranes to release cellular components. Next, the nucleic acids need to be extracted and isolated in a purified solution suited for the downstream steps. This can be done using common analytical techniques such as solid-phase extraction (SPE) or liquid-liquid extraction (LLE) or with more specialised techniques such as using microfluidic-based devices or sequence-specific extraction media. The target nucleic acid, if present, can then be amplified using one of several methods, e.g. polymerase chain reaction (PCR), loop-mediated isothermal amplification (LAMP), recombinase polymerase amplification (RPA), or rolling circle amplification (RCA). Finally, detection can be achieved through a variety of methods with varying degrees of involvement in result interpretation

Similar to conventional tests, there are different targets and thus different options for POC tests, all with their benefits and drawbacks [31]. Out of these, nucleic acid amplification tests (NAATs) stand out due to their high specificity and sensitivity [29]. This is especially important in low endemicity areas, where many other POC tests fail to detect low infection loads [1]. Previously, NAATs were limited to laboratories with specialised equipment and trained personnel, but progress in amplification assays has enabled them to be performed in resource-limited settings as well. However, transferring these tests to the POC requires adaptations due to the relatively complex workflow (see Fig. 1). Therefore, to approach a POC test fulfilling the ASSURED criteria, all steps in the sample-to-answer workflow need to be considered, from sample collection to detection [29, 30, 32–35].

Publications presenting tests for POC settings have primarily focused on the last steps of amplification and detection, giving rise to highly sensitive and specific assays with great potential [29]. However, obstacles such as the requirement for rigorous sample preparation, practicalities around interpretation and reporting of results, and safeguards against cross-contamination have not been as thoroughly discussed. Due to these overlooked aspects in combination with the historical neglect, there are only few examples of NAATs following the ASSURED criteria for NTDs [29, 36].

## Current scope and limitations

**Fig. 2:**
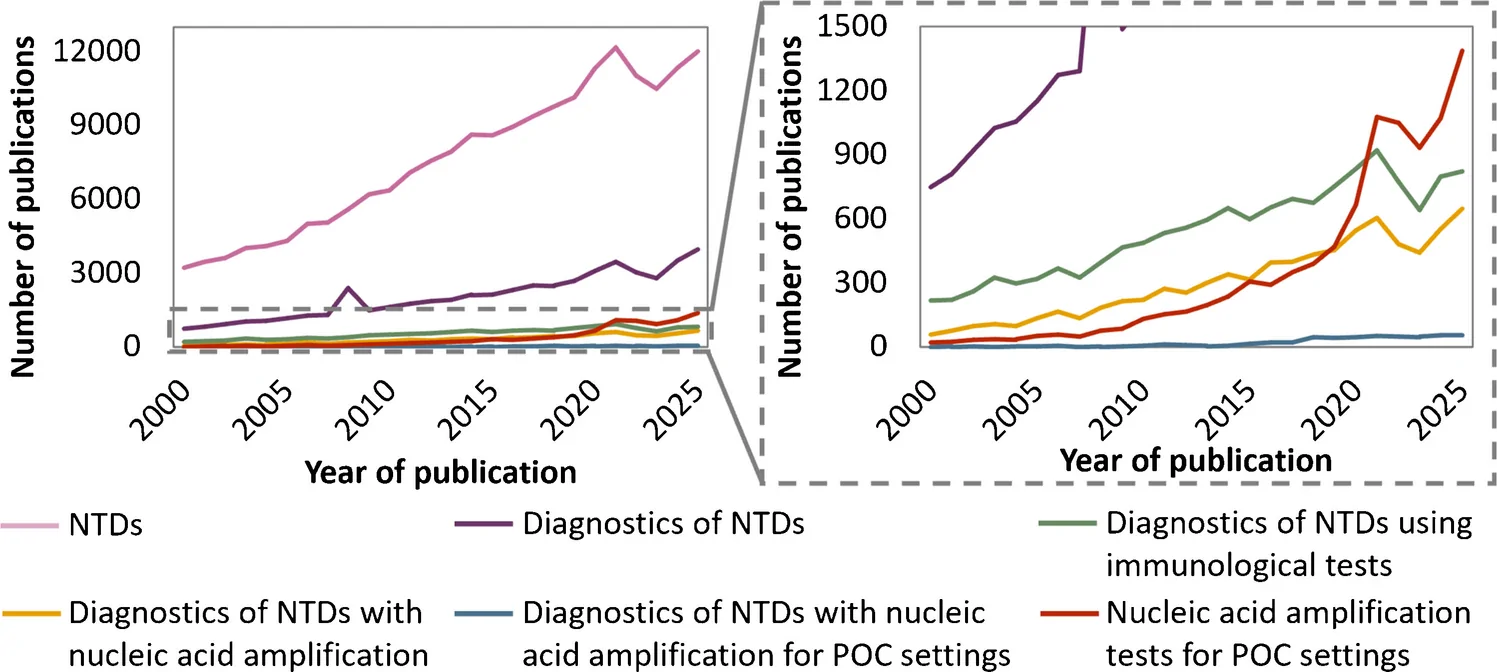
Publication trends related to neglected tropical diseases from 2000 to 2025 based on Scopus data. Total publications found with a search string covering all the NTDs identified by the WHO (pink). Publications found with the search string and the terms “diagnostics or diagnosis or detection” (violet). Publications found with the search string and the terms “diagnostics or diagnosis or detection” and either “immunological or antigen or antibody” (green) or “PCR or qPCR or RT-PCR or LAMP or RT-LAMP or RPA or RT-RPA or RCA” (yellow). Publications found with the search string and the terms “diagnostics or diagnosis or detection”, “PCR or qPCR or RT-PCR or LAMP or RT-LAMP or RPA or RT-RPA or RCA”, and “point-of-care or point-of-need or on site or in field or field-deployable or near POC or decentralised” (blue). Publications found with the terms “diagnostics or diagnosis or detection”, “PCR or qPCR or RT-PCR or LAMP or RT-LAMP or RPA or RT-RPA or RCA”, and “point-of-care or point-of-need or on site or in field or field-deployable or near POC or decentralised” (red)

Based on the identified gaps, the current scope of POC NAATs for NTDs remains constrained. Data from the Foundation for Innovative New Diagnostics test directory underscore this, listing only 17 assays capable of implementation at or near the POC. This limited availability is further reflected in publication trends. Data from Scopus shows a trend of an annual increase in NTD-related articles with more than 10,000 publications yearly since 2019 (Fig. 2). Out of these publications, around 25% per year cover diagnostics of these diseases. Within this subset, NAATs are shown to be less common than for example immunological methods, likely reflecting the earlier establishment and accessibility of immunological tests [37]. Furthermore, when filtering for POC utility, the annual output represents only about 2% of all articles covering NTD diagnostics. This disparity highlights a deficit in research focusing on POC NAATs for NTDs, despite their recognition as an implementation gap for several of these diseases [4]. Furthermore, although POC NAATs in general received low attention in the early 2000 s, the annual publication trend rose rapidly during years of the COVID-19 pandemic which is not as strongly reflected for NTDs. This emphasises that whilst research for POC NAATs is increasing, this is not translated to target NTDs.

To characterise the landscape within this body of literature, this review identifies several overarching trends enabling the shift from centralised nucleic acid amplification tests to POC testing. Moreover, a number of bottlenecks that need to be addressed for deployable tests were also discussed such as the requirement of minimally invasive samples, simplified sample preparation, reduced dependence on power and cold-chain transport as well as increased contamination control, readout objectivity, and validation maturity. Examples of workflows addressing these strategies either in part or as integrated tests are presented in Table 3, with the specific aspects of the workflow and test development outlined in the following sections.

**Table 3:** Reported methods moving towards POC diagnostics of neglected tropical diseases using NAATs

Disease (*pathogen*)	Sample	Extraction	NAAT	Detection	Device/platform	POC angle	Remarks	Ref
**Buruli ulcer *(Mycobacterium ulcerans)***	Swab/tissue	Syringe device	LAMP	Colorimetric	-	Pocket warmer (electricity independent) Simplified extraction Isothermal amplification		[56]
	Swab/tissue	Kit-based	LAMP	Gel electrophoresis	-	Lyophilised reagents Isothermal amplification	Discusses need for simplified extraction	[59]
	Swab/tissue	Closed-cartridge system (syringe)	qPCR	Fluorescence	Biomeme Franklin Mobile qPCR	Lyophilised reagents Field-deployable extraction kit Portable qPCR platform	Field-validated Commercially available Simplified extraction discussed	[60]
**Chagas disease *(Trypanosoma cruzi)***	Genomic DNA	-	LAMP	Conductivity and colorimetric	Paper disc	Low-cost, disposable paper device Isothermal amplification	Sealed device to reduce risk of cross-contamination	[81]
	Blood	Kit-based	LAMP	Fluorescence	-	Prototype LAMP kit for simpler operation Isothermal amplification	Simplified extraction discussed	[50, 68]
	Blood	Kit-based run by 3D printer (PrintrLab)	LAMP	Colorimetric	Repurposed 3D printer (PrintrLab)	Semi-automated extraction and amplification Isothermal amplification	Relatively sophisticated set-up—cost-effectiveness not determined	[84, 85]
	Blood or dried blood spots	Kit-based	LAMP	Fluorescence	-	Rapid extraction Step-by-step protocol Isothermal amplification		[69]
**Dengue virus**	Serum	-	RT-LAMP	Fluorescence	Polyester-toner microdevices	Crude sample Portable equipment Isothermal amplification		[53, 67]
	Serum	Kit-based	RT-LAMP	Potentiometric	Smartphone-connected handheld device	Real-time monitoring using a smartphone app for rapid diagnostics Isothermal amplification	Study partially run in Kaohsiung Medical Hospital in Taiwan	[82]
	Serum	Paper-based	RT-LAMP	Colorimetric	Lab-on-paper	Integrated paper device Isothermal amplification		[42]
	Plasma	Kit-based	RT-PCR	Fluorescence	Portable nucleic acid analyser	Lyophilised reagents Field-deployable device Simple data interpretation from software	Discusses availability of simpler nucleic acid extraction Commercially available device	[62]
**Dengue, chikungunya**	Serum	Paper-based	RT-LAMP	Fluorescence	Lab-on-paper	Integrated paper device Multiplexed detection of viral infectious diseases Isothermal amplification		[70]
	RNA template	-	qRT-LAMP	Fluorescence	Portable LAMP box	Real-time monitoring using a smartphone app for rapid diagnostics Isothermal amplification		[48]
	Blood	Integrated	PCR	Melt curves	BioFire Global Fever Panel	Closed system Minimal hands-on time (2 min) Multiplex Software does the interpretation	Commercially available device Near-POC testing	[89]
**Human African trypanosomiasis**	Blood	Simplified to only lysis	LAMP	Fluorescence	-	Semi-automated process Lyophilised reagents Simplified extraction Isothermal amplification	Bio-inkjet printed kit	[52, 63]
	Template DNA	Kit-based	LAMP	Fluorescence	Digital microfluidic system	False-positive-free detection Low sample volumes Isothermal amplification Portable device	Probe to enhance specificity Closed device	[71]
**Leishmaniasis *(Leishmania major, L. tropica, L. infantum)***	Spiked blood	Kit-based	PCR	LFA	Palm portable PCR machine	Portable thermocycler Simple readout	Field extraction not discussed Thermocycler can be battery operated Commercially available device	[57]
**Leishmaniasis *(L*. *donovani)***	Blood	Kit-based	RPA	Fluorescence	Minoo device	Portable device with smartphone readout Isothermal amplification	Commercially available device	[73]
	Skin punch biopsy	Lysis and magnetic beads	RPA	Fluorescence	Mobile suitcase laboratory Portable fluorescence reader	Rapid extraction Mobile suitcase laboratory Isothermal amplification	Field validated	[66]
**Leishmaniasis (*Leishmania* spp.)**	Parasite	Detergent-based lysis	RPA	Fluorescence	-	Instrument-free Sample preparation suitable for resource-limited settings Isothermal amplification	Next steps lyophilising reagents Prototype for integrated device	[74]
**Leprosy *(Mycobacterium leprae)***	Tissue	Simplified protocol	qPCR	Fluorescence	Q3-Plus	Portable thermocycler with real-time monitoring Simplified workflow	Commercially available device	[75]
**Lymphatic filariasis *(W. bancrofti, Brugia malayi)***	Blood	On-chip trapping Kit-based	PCR	LFA	Semiautomated microfluidic device miniPCR	Portable thermocycler		[87]
**Mycetoma *(Madurella mycetomatis, Falciformispora senegalensis)***	Fungal and bacterial isolates of causative agents	-	RPA	Fluorescence	-	Multiplex detection Isothermal amplification		[76]
**Rabies**	Brain tissue	Kit-based	RT-qPCR	Fluorescence	PicoGene PCR1100	Designed for rapidity, mobility, and practicality		[77]
**Schistosomiasis *(Schistosoma haematobium)***	Urine	Heating	LAMP	Colorimetric	-	Crude samples possible to use Isothermal amplification	Tested in a resource-poor laboratory in endemic area	[43, 44]
	Stool, urine, blood, swab	Rapid kit-based for resource-poor settings	LAMP	Fluorescence	T-cup	Instrument-free Electricity-independent Isothermal amplification		[58]
	Urine	Simplified kit	RPA	Fluorescence	Portable fluorometer	Extraction suitable for resource-poor settings Isothermal amplification		[78]
	Swab	Simplified kit	RPA	Fluorescence	-	Extraction suitable for resource-poor settings Isothermal amplification	Home-based self-swabs might be possible	[79]
	Urine	Two-step kit	RPA	Fluorescence	-	Cold-chain independent Extraction suited for resource-limited settings Isothermal amplification Blue light can be used instead of fluorometer		[47]
**Schistosomiasis *(S. mansoni)***	*Schistosoma* adult worms	Kit-based	LAMP	Colorimetric and fluorescence	Handheld portable device	Cold-chain independent User-friendly smartphone app Isothermal amplification		[83]
	Whole blood	On-chip with kit-based reagents	LAMP	Fluorescence	Microfluidic cassette	Field-ready microfluidic device Equipment-free Isothermal amplification		[54]
**Soil-transmitted helminth infections *(Trichuris trichiura)***	*Trichuris trichiura* worms	Kit-based using automated system	PCR	Distance-based using intercalating dye	Paper-device	Low-cost device	Discusses integrated DNA extraction and isothermal amplification as next steps	[90]
**Taeniasis *(Taenia solium)*, foodborne trematode infection *(Clonorchis sinensis)***	Stool, tissue	Kit-based	LAMP	Fluorescence	Dished microfluidic chip	Multiplexed detection Isothermal amplification		[80]
**Trachoma *(Chlamydia trachomatis)***	Swab	Off-chip multistep	LAMP	LFA	Paper-device	Lyophilised reagents Simple chip Isothermal amplification	Field validation	[61]

### Sample type

The publications listed in Table 3 aim to develop diagnostic tests suitable for resource-limited settings at the POC. However, most of these articles only target part of the NAAT workflow. Out of the presented studies, the majority (32/39) validated their tests on spiked or clinical samples. For POC tests, the sample should ideally be minimally invasive and easy to collect, such as saliva, urine, swabs, or capillary blood from a finger prick [28]. Depending on the disease, this can be limiting. One reason for this is that the target amount that can be collected is in part dependent on the sample type for infectious targets [38]. A transition to a non-invasive sample could therefore lower the sensitivity of the test, especially without a concentration step. Different specimens also introduce their own biochemical challenges due to the different matrices and present interferents [39]. For example, blood contains several endogenous inhibitors such as haemoglobin and immunoglobulin G which puts requirements on robust extraction or inhibitor-resistant enzymes [40]. Conversely, alternative matrices such as urine introduce additional challenges that stem from exogenous inhibitors with variables such as diet in addition to inhibitors such as urea. Moreover, the sample type determines the necessary pre-analytical considerations, such as timing of collection and sample container additives. Environmental variables like ambient temperature and humidity are also critical factors that impact sample stability and consequently assay performance. Since POC diagnostics in NTDs are mainly needed in resource-limited settings, this comes with logistical constraints [1]. This is mostly related to the desired lack of cold-chain infrastructure and minimum-infrastructure settings which can compromise the sensitivity and specificity of the assay [28, 40]. Consequently, the sample type alone comes with several important considerations to achieve a viable POC NAAT.

In the reviewed articles, the most common sample type was blood which can be invasive depending on whether a finger prick or a venous blood draw is required. Whilst venous blood would yield a more accurate test than capillary blood, it is invasive and requires trained personnel which is not ideal [41]. For blood samples, the choice of matrix between whole blood, serum, or plasma will also determine of the sensitivity and necessary sample preparation [28, 34, 35, 38, 41, 42]. Moreover, a switch from venous blood to capillary blood restricts the sample volume which directly limits the analytical sensitivity putting higher requirements on the test [38]. The possible clinical samples that can be used for the test will therefore play a role in the assay protocol, sensitivity, and suitability based on the ASSURED criteria [28, 38].

### Sample preparation

Once the sample is collected, the usually most hands-on intensive part of the workflow follows, namely the sample preparation [36]. This part includes the steps from cell lysis to nucleic acid purification and concentration. Traditionally, this is performed using commercially available kits [34]. However, these kits drive up the cost of the tests and often require instrumentation such as centrifuges which does not align with part of the REASSURED criteria since it makes the assays less affordable and accessible [36].

Some alternatives have been presented that range from direct crude sample addition to simplified lysis and purification [43, 44]. Using crude samples would be preferable to reduce the time to result, the manual work steps, and the cost of the test. However, it also comes with challenges. These mainly pertain to the presence of previously mentioned inhibitors, such as haemoglobin and urea, that interfere with amplification for example by disrupting enzyme activity giving rise to false-positive and false-negative results [34, 36]. The extent of this reduction of amplification efficiency varies for the different amplification assays with PCR being more susceptible to this than isothermal methods such as loop-mediated isothermal amplification (LAMP) or recombinase polymerase amplification (RPA) [45, 46].

Two of the reviewed articles employ these sample-preparation-free methods, one using RPA for schistosomiasis diagnosis with a spiked urine sample and the other reverse transcription LAMP (RT-LAMP) for Zika diagnosis with human urine or saliva [47, 48]. For these examples, a critical aspect for circumventing the extraction is the sample volume. A higher volume increases the probability that pathogen DNA or RNA is present, but it also increases the inhibition risk. Additionally, highly sensitive detection is required since there is no concentration step [38]. As an example, the direct amplification from spiked urine for schistosomiasis showed that it is possible to detect a single *Schistosoma haematobium* egg in 6 µL of urine. However, assuming a homogeneous distribution of eggs in the sample the infection load would therefore need to be very high, considering the definition of low infection loads being less than 50 eggs per 10 mL urine [47, 49]. Since one of the advantages of using NAATs is the sensitivity, the usefulness of an assay without the sample preparation should be critically considered if it yields low sensitivity, especially since the more common alternative of immunological testing inherently requires minimal to no rigorous sample preparation [38].

Apart from the use of crude samples, there are also examples of simplified protocols that do not require kits. These can roughly be divided into three categories for the presented publications, namely (I) thermal lysis, (II) instrument-free, as well as (III) integrated and paper-based extraction. Firstly, the thermal lysis methods can be described as *boil and spin* where the heating lyses the cells and centrifugation removes the cell debris for a simple extraction. Even though this solution does not remove the need for equipment, it does contain fewer steps and largely reduces the reagent costs with these being designed for resource-limited settings [43, 44, 50]. Moreover, examples apart from bulky and electricity-dependent centrifuges exist such as hand-powered centrifuges, centrifugal microfluidic solutions, and paperfuges which could simplify the centrifugation steps [51]. Secondly, an instrument-free method employed a lysis buffer with detergents and then used the lysate for amplification making it rapid and simple [52]. Lastly, there are also protocols for integrated extractions on devices with off-chip lysis followed by trapping of nucleic acids on different types of membranes or beads and wash steps. These methods follow the conventional workflow of NAATs with lysis, purification, and concentration but without the need for commercial kits [42, 53, 54]. Apart from these examples, sample preparation remains overlooked in many of the reviewed articles, where the focus is instead on user-friendly amplification and detection [29, 36, 38].

### Nucleic acid amplification and reagent stability

The amplification methods in the publications presented in Table 3 are all described as suitable for decentralised testing using either the gold standard PCR or one of the more widely used isothermal methods. PCR has conventionally been limited to laboratories with benchtop thermocyclers and has therefore not been suited for POC tests [46]. With the current portable thermocyclers, however, PCR can arguably be used for POC tests [55]. Nevertheless, this is not fully in line with the ASSURED principle since specialised equipment is required [27, 28]. Isothermal amplification also requires a heat source, but since it operates at constant temperatures, this does not need to be sophisticated [46]. In addition to an already simple setup with a hot plate, there are also examples of electricity-independent workflows. These include using battery-operated heat sources [56, 57] and a less conventional setup using modified coffee capsules in a water bath [58]. The low requirement for laboratory instruments means that these should be more readily implemented as POC tests [56–58].

Even with the availability of amplification methods that can be performed with simple instrumentation, a commonly cited hindrance for employing these in resource-limited settings is the cold-chain dependence of the reagents. This includes storage but also, more importantly, transport. Transport with cold packs or dry ice drives up the cost in addition to the already expensive reagents, which in turn drives up the cost of each test. To limit this effect, several publications used lyophilised reagents or protocols to lyophilise them. This together with user-friendly detection is an approach seen in several of the articles that aim for POC tests in resource-limited settings, but these do not all consider a simplified extraction [52, 59–63]. Moreover, despite overcoming the cold-chain hurdle, difficulties such as environmental factors (e.g. humidity) on the reagents as well as shelf life remain, which show the importance of field validation [64].

### Detection strategies

The last step in the bioanalytical workflow, detection, comes with several alternatives that range from low-cost, instrument-free qualitative readouts to highly sensitive and specific, instrument-dependent quantitative assays [65]. In the reviewed articles, the most common detection assay was fluorescence [47, 48, 50, 52, 54, 60, 62, 63, 66–80]. This offers high sensitivity and specificity as well as real-time, quantitative monitoring using either sequence-specific probes or intercalating dyes. These characteristics align with most of the performance aspects of the ASSURED criteria, but the need for instrumentation does not. To circumvent this requirement, portable instruments and handheld lights combined with optical filters have been suggested as simpler solutions. Moreover, whilst intercalating dyes are the less expensive option, the more costly alternative of detection using sequence-specific probes comes with advantages of both higher specificity and allows for multiplexing and detection of co-endemic pathogens simultaneously.

Alternatives in the form of electrochemical sensors that do not require optical equipment have also been presented in the reviewed articles [81, 82]. These two examples differ in what is detected, but they share a common drawback which is that the detection is indirect. Instead of verifying the target, the detection is based on by-products of the reaction such as shifts in conductivity or proton accumulation. A potential limitation of this is that the effect of non-specific amplification or buffered sample matrices could either trigger or suppress this signal, lowering the specificity and sensitivity of the test.

This vulnerability also holds for colorimetric assays, albeit with the advantages of instrument-free detection which is suitable for resource-limited settings [42–44, 56, 83–85]. Colorimetric assays typically rely on pH or metal-ion indicators that drive a colour change. Since these indicators track chemical byproducts, the readout is not intrinsically amplification-dependent [86]. Consequently, these assays become susceptible to false positives or negatives due to the buffering capacity of samples or background contamination that can suppress or enhance the colour change.

Lateral flow assays (LFAs) present a fourth alternative that overcomes the previously mentioned limitations by using paper-based immunochromatography to capture labelled amplicons [57, 61, 87]. However, in the context of NAATs, tubes need to be opened after amplification to dip an LFA test strip. This action can release amplicons into the air, posing a risk of cross-contamination and compromising long-term use unless open tubes can be avoided through more innovative approaches [46].

Ultimately, for application to NTD diagnostics, the high sensitivity of NAATs is a strong advantage [29]. However, the requirements for detection will depend on the identified levels for low infection loads for the different conditions. Comparing, for example, detection of *Schistosoma mansoni* for diagnostics of schistosomiasis and the detection of *Ascaris lumbricoides* for diagnostics of soil-transmitted helminths the limits are < 100 eggs per gramme of stool and < 5000 eggs per gramme of stool, respectively [88]. Hence, due to the diversity of causative agents for NTDs, the comparison of test requirements becomes difficult to perform across the entire group [1].

### Total assay time

An additional consideration for POC NAATs, apart from optimising the individual steps, is the total assay time. Due to the many steps of a NAAT workflow, these tests can take up to several hours to perform which limits the utility in POC settings where short turnaround times are preferred to ensure timely clinical intervention [29, 35]. Despite this, the majority of the reviewed articles did not present this metric. The reported assay times were instead mainly focused on the amplification step rather than the entire process, even though sample preparation often represents a considerable part of the total time [34]. To meet the established criteria for rapid POC tests, however, the total assay time is an important factor to consider [28].

### Examples of test set-ups and workflows

The articles in Table 3 all have adjustments of the conventional NAAT workflow that make the tests more suitable for POC use. These modifications can be linked to either simplifying the workflow, using a set-up with modified instruments, or integrating steps on microfluidic devices. Examples showcasing these different approaches are discussed below.

**Fig. 3:**
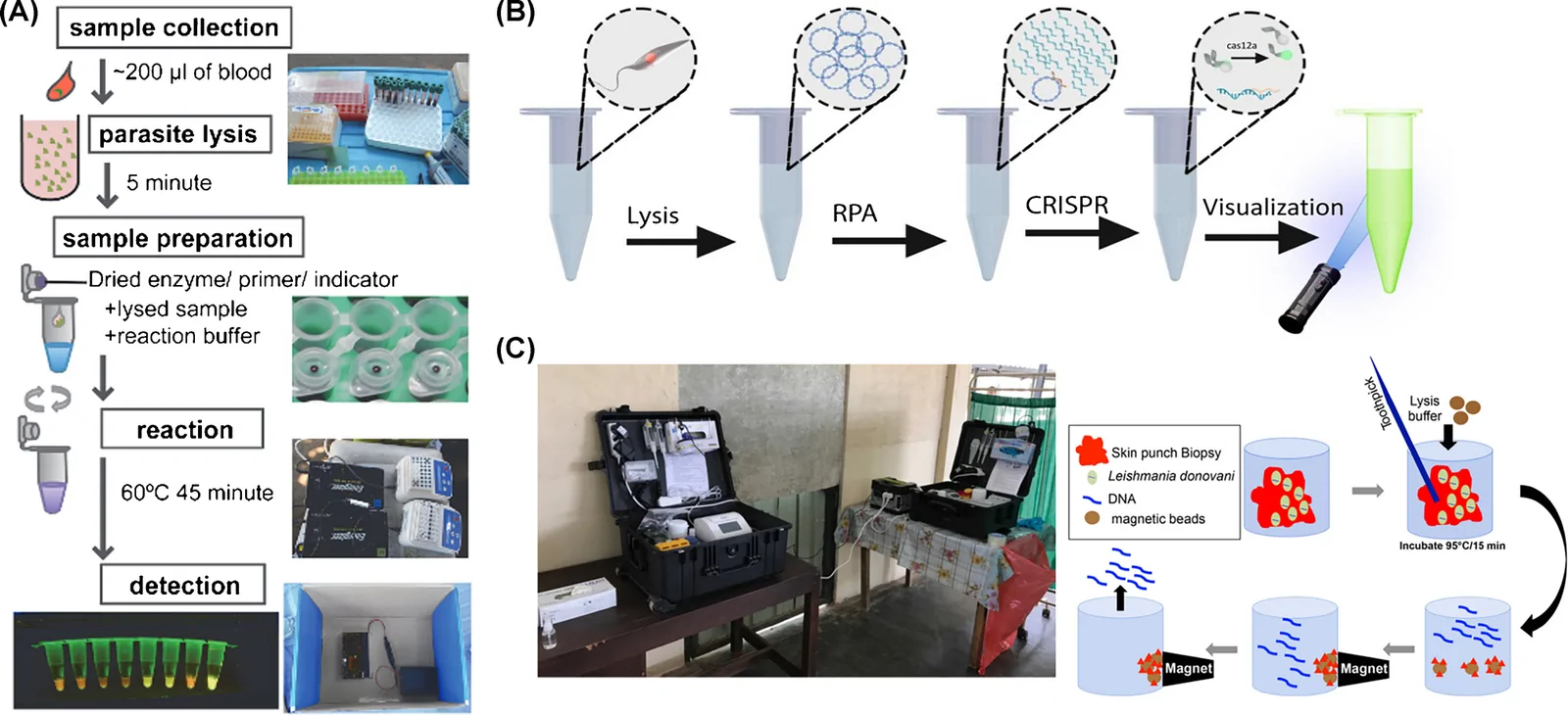
Approaches to POC NAATs for NTD diagnostics using simplified workflows for **A** human African trypanosomiasis and **B** leishmaniasis. Figures adapted from references [52] and [74], respectively, open access. **C** A suitcase laboratory with a simplified extraction method for detection of leishmaniasis. Figure adapted from ref. [66], open access

Simplifying the requirements of the assays has been attempted by moving towards isothermal amplification. However, for a suitable POC test, one also needs to consider the nucleic acid extraction [38]. Three examples of this are shown in Fig. 3. Two of these (Fig. 3A, B) leverage the fact that LAMP and RPA are less prone to inhibition and only use detergent lysis [52, 74]. Moreover, both of these assays also address cold-chain dependence by either using lyophilised reagents [52] or by investigating strategies to lyophilise reagents [74]. Despite this, the detection still requires instrumentation with transilluminators which increases the cost. The third example of simplified extraction, albeit with a kit, is a suitcase laboratory with consumables and instruments for all steps in the workflow that was validated for use at the POC (Fig. 3C) [66]. This showed that compromises such as lower sensitivity might be required as this can be a consequence of rapid extraction compared to conventional workflows. Moreover, an invasive sample from punch biopsies was also a drawback of this workflow since these need to be performed by highly trained personnel. A less invasive alternative that was presented is skin scrapings; however, this lowered the sensitivity further. Consequently, an alternative sample collection method was identified as future work to make this suitcase laboratory fully suited for field-based diagnostics.

**Fig. 4:**
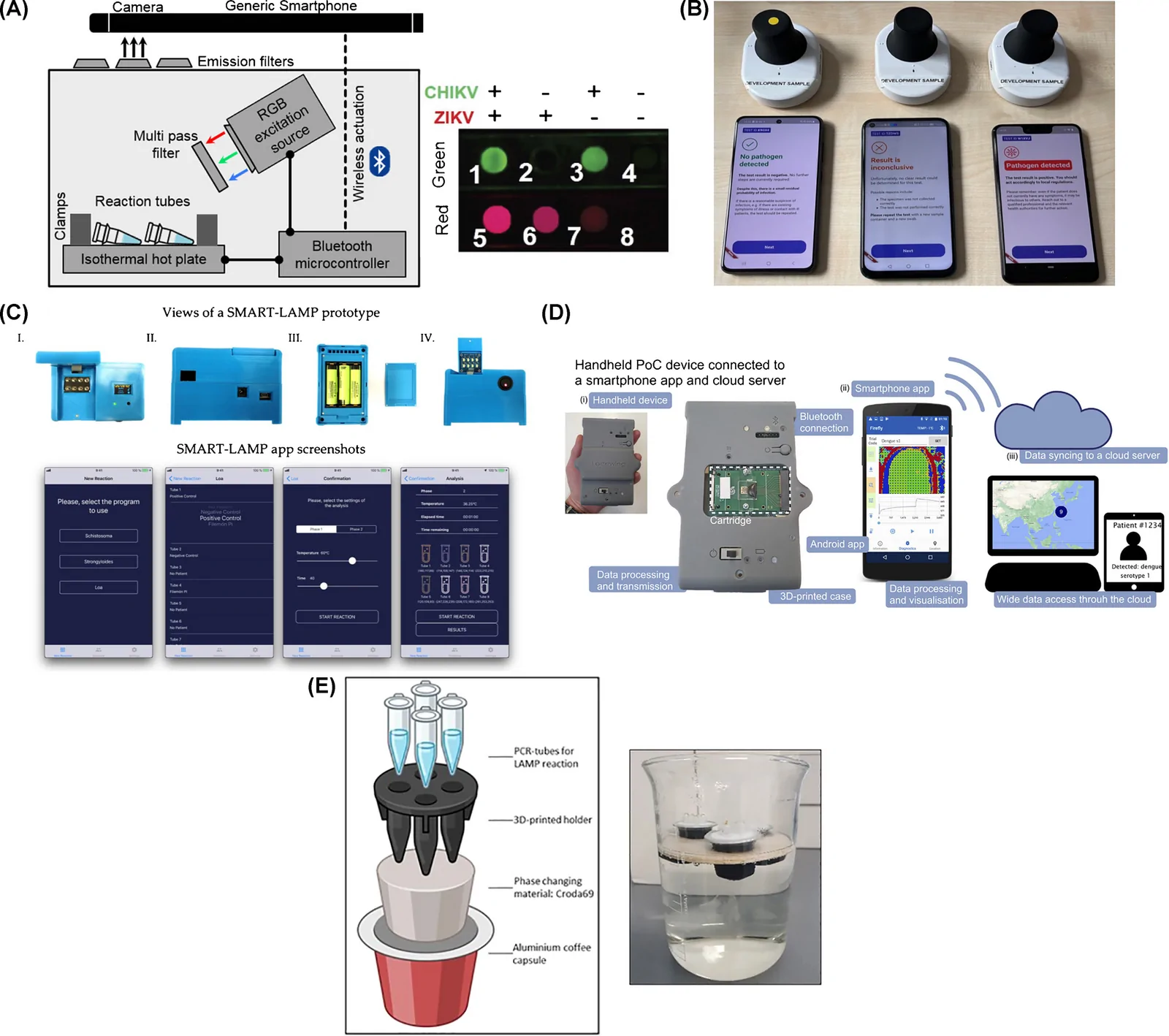
Approaches to POC NAATs for NTD diagnostics using modified or developed instruments. **A** A smartphone-connected detection module for multiplex detection of Zika and chikungunya virus. Figure adapted from ref. [48], open access. **B** A portable, smartphone-connected instrument for amplification and detection, here used for detection of leishmaniasis. Figure adapted from ref. [73], open access. **C** Smartphone-connected handheld device for LAMP, here for the detection of schistosomiasis. Figure adapted from ref. [83], open access. **D** Handheld lab-on-a-chip platform for detection of Dengue virus. Figure adapted from ref. [82], open access. **E** An instrument-free amplification using modified coffee capsules for detection of schistosomiasis. Figure adapted from ref. [58], open access

Additionally, there are examples in the literature where instruments are modified or developed to make the tests portable for POC applications (Fig. 4). In Fig. 4A, a LAMP box is shown that can be used for multiplexed detection of chikungunya and Dengue virus in crude samples using different fluorophore labels [48]. This workflow was developed with considerations to POC suitability from sample collection to detection and interpretation, even though, as the authors discuss, this comes with limitations in regard to sample volume and sensitivity. Other portable devices are the minoo prototype for *Leishmania* detection (Fig. 4B) [73], SMART-LAMP for diagnosis of schistosomiasis (Fig. 4C) [83], and Lacewing for Dengue detection (Fig. 4D) [82], which are battery-driven devices with fluorescence, colorimetric, and electrochemical detection, respectively. All these have wireless connections to smartphone apps with immediate readout aiding in the real-time connectivity component of the REASSURED criteria. These setups for amplification and detection are designed with simplicity in mind as well as electricity independence. However, kit-based extraction still imposes requirements on laboratory instruments which the authors identify as a limitation to address in future work [73, 82, 83]. Lastly, repurposed coffee capsules filled with biobased material act as heaters in a water bath for amplification before naked eye detection (Fig. 4E) [58]. This setup was designed with cost-effectiveness, scalability, and low environmental impact in consideration [91]. However, it was only validated with a limited number of known positive samples divided over two runs with varying results. In both runs, sensitivity became limiting, possibly due to time constraints of the heating [58].

**Fig. 5:**
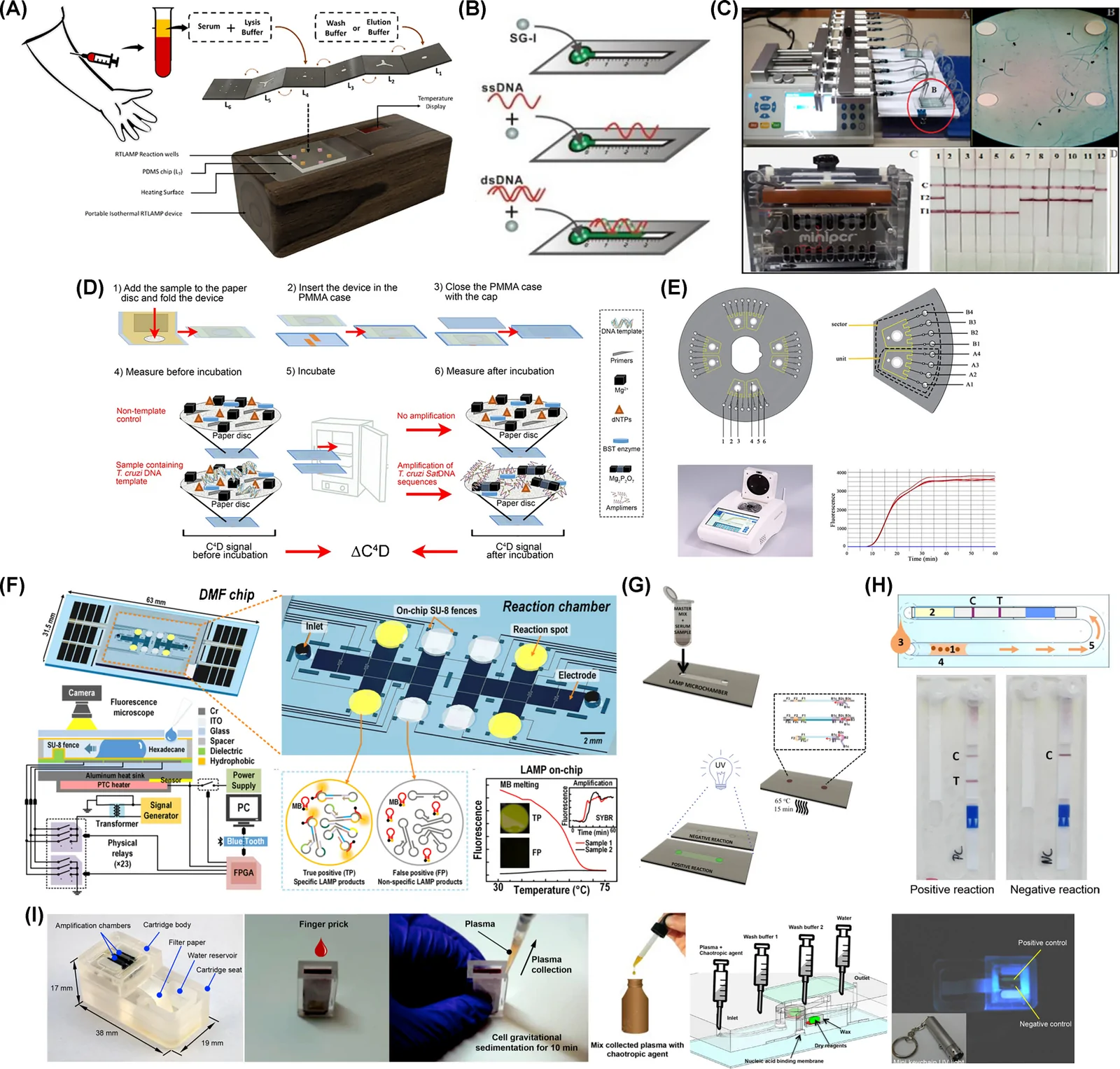
Approaches to POC NAATs for NTD diagnostics using microfluidic devices with varying degrees of integrated workflows. **A** An integrated workflow on a multi-layered paper/polymer-based device for detection of dengue virus. Figure adapted from ref. [42] with permission from the American Chemical Society. **B** A paper-based device for distance-based quantification of soil-transmitted helminths. Figure adapted from ref. [90] with permission from the American Chemical Society. **C** A portable thermocycler coupled with LFA for detection of lymphatic filariasis. Figure adapted from ref. [87], open access. **D** Disposable paper disks with contactless conductivity readout using a reusable plastic housing for diagnosis of Chagas diseases. Figure adapted from ref. [81], open access. **E** Dished microfluidic platform for multiplex detection of parasites including Taenia solium. Figure adapted from ref. [80], open access. **F** A digital microfluidic system for amplification and specific detection of human African trypanosomiasis. Figure adapted from ref. [71], open access. **G** An integrated workflow using disposable polyester-toner microdevices. Figure adapted from ref. [67], open access. **H** A closed system chip with lyophilised LAMP reagents and a lateral flow strip for detection of trachoma. Figure adapted from ref. [61], open access. **I** An instrument-free, integrated workflow using microfluidic cassettes for detection of schistosomiasis. Figure adapted from ref. [54], open access

Moreover, there are also attempts to use microfluidic platforms to integrate or simplify the workflow. One approach is via paper-based devices, with an example shown in Fig. 5A. These devices utilise the folding or stacking of paper layers to process samples before amplification by capillary flow through lysis and wash buffers to isolate nucleic acids. This principle has been shown for both single and multiplexed detection of pathogens for POC tests [42, 70]. Another approach is a distance-based paper device that aims to simplify the quantitative detection (Fig. 5B) [90]. Despite using conventional PCR, a portable thermocycler and a pocket-size light box were used in their report to limit the use of bulky instrumentation. Moreover, it shows potential for implementation with isothermal amplification, which would further reduce the need for specialised instrumentation.

Apart from these paper-based devices, there are also other microfluidic platforms that have been developed with one or more on-chip steps. A first example of this is a setup for diagnosis of lymphatic filariasis with a microfluidic chip connected to pumps for isolation of parasites and a battery-driven thermocycler (Fig. 5C) [87]. This protocol reduces the time to result as compared to the gold standard and can handle multiple samples at once which presents advantages for POC tests. However, bulky equipment is required for this test, including pumps, a centrifuge, and a thermocycler which may complicate the implementation at the POC. A second assay that also requires several instruments is a disposable paper disc in a reusable plastic case for diagnosis of Chagas disease [81]. This differs from the other workflows in that no optical equipment is needed, but it does not remove the instrumentation entirely. Moreover, a test with clinical or spiked samples was not carried out and potential requirements of extraction was not evaluated but mentioned as a next step (Fig. 5D). The third example also did not include integrated sample preparation but reduced the instrumentation needed for multiplexing, amplification, and readout to a disk with four sectors each consisting of four reaction chambers and a device with integrated modules [80]. Whilst reducing the number of instruments, it does require a specialised instrument (Fig. 5E). Another technologically sophisticated example with off-chip extraction is shown in Fig. 5F that uses a digital microfluidic system for *Trypanosoma* detection [71]. This workflow showed several advantages for the amplification part with reduced reagent consumption and closed vessel quality control which reduces the risk of aerosol contamination. For the other major steps, integrating the chip with detection and sample preparation modules was suggested to make this more suited for POC tests. Contrary to these workflows with off-chip extraction, an example of a device with detection directly from serum is a custom polyester-toner microdevice for rapid detection of Dengue virus (Fig. 5G) [67]. This does simplify the workflow to a single step, but the presence of inhibitors in the crude samples resulted in a higher limit of detection compared to purified samples. Although not used in this article, a developed extraction protocol for this same chip may offer a solution for an integrated workflow to achieve better sensitivity [53, 67]. Another rather user-friendly device involved a simplified sample preparation narrowed down to only lysis paired with a chip that contained lyophilised amplification reagents and a lateral flow strip readout (Fig. 5H) [61]. Whilst this shows consideration of the full workflow, limitations such as low sensitivity due to inhibition and aerosol contamination remain. Lastly, an example of a fully integrated device is a disposable cassette for schistosomiasis detection that integrates purification, concentration, amplification, and detection (Fig. 5I) [54]. This cassette holds a membrane for trapping of nucleic acids in a chamber with encapsulated amplification reagents that are released upon heating. The presented design allows large volumes of sample to be filtered, concentrated, and amplified for POC testing with high sensitivity. Overall, these examples show the potential of portable and integrated diagnostic tests, characteristics that make them suitable for POC tests in resource-limited settings [92]. However, in addition to the previously mentioned barriers, these platforms come with additional hindrances related to the design and manufacture of devices. A focus on the cost, reproducibility, and complexity rather than innovation is therefore important to achieve a deployable platform [92–94].

## Strengths, weaknesses, opportunities, and threats

**Fig. 6:**
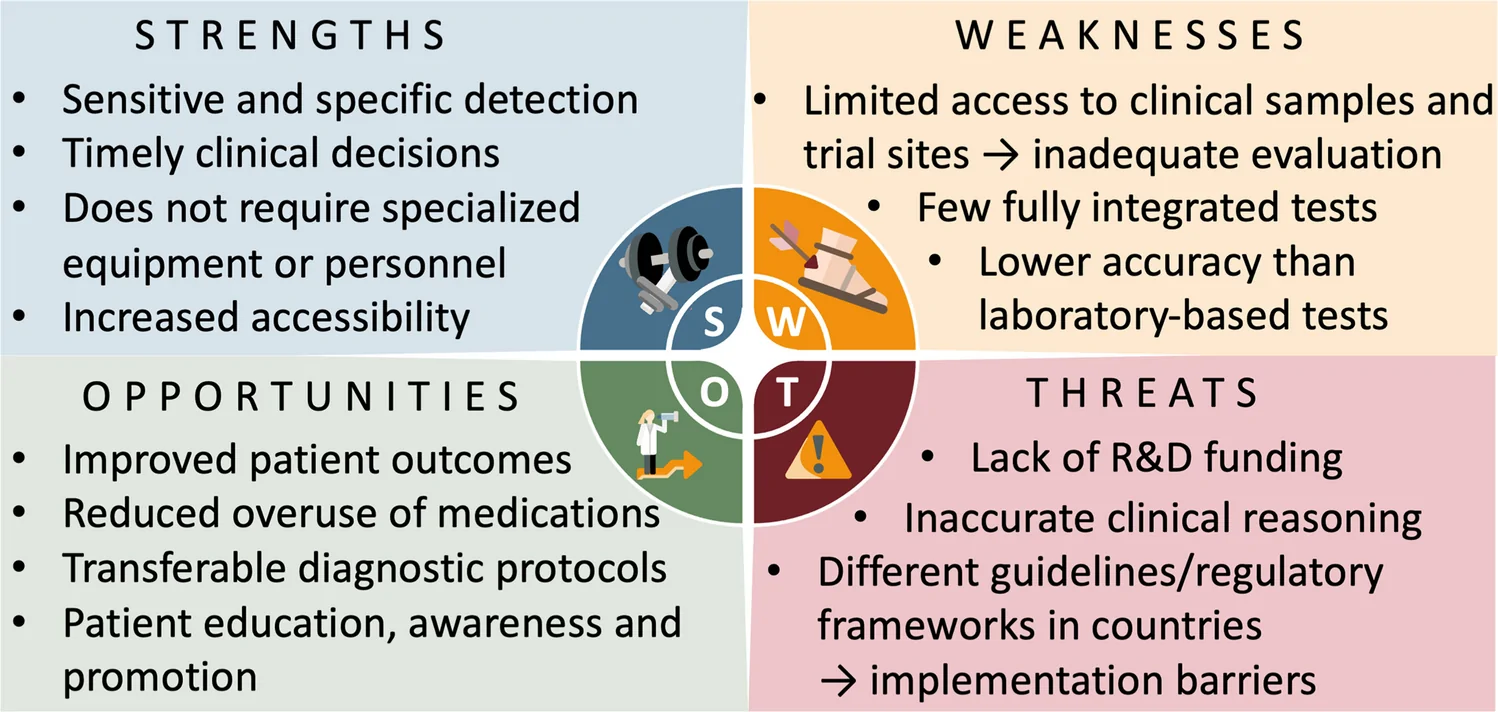
SWOT analysis of POC NAATs for diagnostics of NTDs

Development of POC diagnostic tests for NTDs has historically not been an R&D priority. Consequently, the numbers of reports on NAATs for this group of over 20 diseases remain low in the literature. However, with the identified need for diagnostic tests for NTDs and the nearing deadline of the sustainable development goals, several of which depend upon control of NTDs, the historical neglect should be alleviated [1]. This should in turn lead to increased interest in finding solutions for effective, standardised, and affordable diagnostics, a field where NAATs show several advantages (see Fig. 6) [64].

Current NAATs do not necessitate expensive and bulky equipment such as benchtop thermocyclers since both isothermal amplification methods and portable thermocyclers have been developed. This reduces the power consumption. Isothermal methods also offer opportunities by allowing the handling of semi-crude or crude samples better than the conventional PCR [45, 46, 64]. Additionally, these isothermal NAATs show superior sensitivity and specificity compared to rapid immunological tests, which is crucial for detecting low infection loads and interrupting transmission [33]. Moreover, this shift from centralised to decentralised NAATs supports more rapid turnarounds for timely clinical decisions. Altogether, this increases the equity with respect to sensitive and specific diagnostic access for marginalised populations in resource-limited settings [28].

As with other developing research areas, there are weaknesses as well. Firstly, even though there are several academic prototypes, the limited access to clinical samples and trial sites in endemic regions makes it difficult to validate tests in the intended setting [28]. Secondly, the requirement of costly kit-based sample preparation limits the applicability. Moreover, even with several examples of microfluidic devices for more user-friendly workflows, developing fully integrated sample-to-answer platforms remains a challenge [64]. Lastly, there is the risk of lower diagnostic accuracy compared to centralised laboratory testing. However, it is important to note that a POC test can still be the better option, as the most accurate test does not always equal the most useful test. This stems from high-accuracy tests being limited to centralised laboratories, which has been shown to be infeasible for surveillance of NTDs in resource-limited settings [95].

Apart from these technical aspects, there are large-scale opportunities for successful deployment of POC NAATs. For example, by leveraging previous research in POC testing for other pathogens, researchers can access translatable workflows that streamline NTD diagnostics through an increased readiness for field deployment [96–98]. Transitioning to more widespread POC testing enables a more extensive transmission mapping whilst simultaneously moving away from dependence on mass drug administration and centralised testing. This in turn allows for targeted deployment of resources where they are needed most, rather than relying on inefficient treatments [1, 8]. Moreover, the availability of NAATs at the POC would mean that previously low burden infections can be diagnosed which could prevent the progression to severe, often irreversible sequelae of NTDs by ensuring timely intervention [1, 30]. Ultimately, the availability of sensitive and specific NAATs at the POC has the power to educate patients about transmission routes and prevention strategies, which can increase trust in healthcare systems due to the direct link between testing and care [30].

Despite these opportunities that make POC NAATs promising, several threats or obstacles make the transfer from research to clinical use difficult. Firstly, the lack of R&D funding for diagnostic tests for NTDs [99]. Consequently, few scalable devices for NAATs that are deliverable to end-users exist [94]. Secondly, although one benefit of POC tests is that they do not require highly trained personnel, this comes with risks such as inaccurate clinical reasoning. For instance, this can be problematic for false-positive results where reasoning about the alignment of symptoms and clinical presentation becomes important [30]. Lastly, multiple endemic countries entail multiple regulatory frameworks and guidelines which makes standardisation less straightforward for novel diagnostics. Therefore, to achieve the full potential of these tests, all aspects and not just the experimental workflows, need to be considered [100].

## Conclusion

Given that the WHO has identified the availability of diagnostic tests in resource-limited settings as a critical need for several NTDs, the transition of NAATs from centralised laboratories to the POC represents a promising strategy. The adoption of isothermal amplification methods, particularly LAMP and RPA, has demonstrated that high analytical sensitivity can be achieved without the requirement of specialised equipment in the form of thermocyclers, lowering the barrier for implementation of molecular diagnostics. However, the transition to practical POC tests remains incomplete due to persistent gaps, including costly and time-consuming sample preparation, cross-contamination risks, cold-chain dependence, and logistical implementation barriers as well as a lack of validation in endemic environments. The overall lack of R&D towards the manufacture of mature and validated tests that are fit for the field continues to halt many developments at a proof-of-concept stage. To move past this, both analytical and operational barriers should be addressed to reach decentralised diagnostic tools. Moreover, translating more mature diagnostic platforms developed for other pathogens to NTD diagnostics can help bridge these gaps, ultimately producing tests that align with the ASSURED criteria and supporting the NTD targets set by the WHO for 2030.

## Data Availability

No data were used for the research described in the article.
